# Age-specific oxidative status and the expression of pre- and postcopulatory sexually selected traits in male red junglefowl, *Gallus gallus*

**DOI:** 10.1002/ece3.300

**Published:** 2012-07-27

**Authors:** Jose C Noguera, Rebecca Dean, Caroline Isaksson, Alberto Velando, Tommaso Pizzari

**Affiliations:** 1 Dpto. Ecoloxia e Bioloxía Animal, Edificio de Ciencias Experimentales, Universidad de Vigo36310, Vigo, Pontevedra, Spain; 2 Edward Grey Institute, Department of Zoology, University of OxfordOxford, OX1 3PS, UK; 3 Department of Evolutionary Biology, Evolutionary Biology Centre, Uppsala UniversityUppsala, Sweden; 4 Department of Biology, University of LundSölvegatan 37, 223 62, Lund, Sweden

**Keywords:** Oxidative stress, reproductive restraints, reproductive senescence, sexual selection, sperm competition

## Abstract

Oxidative stress is emerging as a key factor underpinning life history and the expression of sexually selected traits. Resolving the role of oxidative stress in life history and sexual selection requires a pluralistic approach, which investigates how age affects the relationship between oxidative status (i.e., antioxidants and oxidative damage) and the multiple traits contributing to variation in reproductive success. Here, we investigate the relationship between oxidative status and the expression of multiple sexually selected traits in two-age classes of male red junglefowl, *Gallus gallus*, a species which displays marked male reproductive senescence. We found that, irrespective of male age, both male social status and comb size were strongly associated with plasma oxidative status, and there was a nonsignificant tendency for sperm motility to be associated with seminal oxidative status. Importantly, however, patterns of plasma and seminal antioxidant levels differed markedly in young and old males. While seminal antioxidants increased with plasma antioxidants in young males, the level of seminal antioxidants remained low and was independent of plasma levels in old males. In addition, old males also accumulated more oxidative damage in their sperm DNA. These results suggest that antioxidant allocation across different reproductive traits and somatic maintenance might change drastically as males age, leading to age-specific patterns of antioxidant investment.

## Introduction

Oxidative stress occurs when antioxidants defenses cannot fully compensate for the oxidant activity of reactive molecules (i.e., reactive oxygen species, ROS; Halliwell and Gutteridge 2007), and is thought to be a universal cause of aging and a fundamental factor in life history evolution (Finkel and Holbrook 2000). Recent work suggests that oxidative stress might be particularly relevant to male traits affecting reproductive success and targeted by sexual selection (von Schantz et al. 1999; Dowling and Simmons 2009; Monaghan et al. 2009). Sexual selection operates on traits that affect a male's ability to compete for mates, such as ornaments and competitive ability, and those that affect the ability of his ejaculates to compete over fertilization, when females mate multiply (Birkhead and Møller 1992; Andersson 1994; Pizzari and Parker 2009). Oxidative stress might mediate the condition-dependent expression of sexual ornaments, via antioxidant allocation trade-offs (von Schantz et al. 1999; Blount et al. 2003; Pike et al. 2007; Pérez et al. 2008; Dowling and Simmons 2009; Monaghan et al. 2009), and has also been identified as an important factor shaping the fertilization efficiency of an ejaculate under sperm competition (Blount et al. 2001; Poiani 2006; Velando et al. 2008; Dowling and Simmons 2009; Almbro et al. 2011). For example, sperm motility and viability can be affected by the antioxidant capacity of seminal fluid, which protects sperm from oxidative damage (Poiani 2006; see also den Boer et al. 2010; Simmons and Beveridge 2011). Oxidative stress deteriorates sperm plasma membrane and DNA, leading to a decline in fertilizing efficiency and sperm competitiveness (Aitken and Baker 2006; Poiani 2006).

As their physiological performance declines (Rose 1991), aging organisms may suffer from less efficient antioxidant systems, and as a consequence, become more vulnerable to oxidative stress (Sohal and Weindruch 1996; Finkel and Holbrook 2000; Torres and Velando 2007). Therefore, antioxidant demands exacted by reproductive investment may be become relatively more costly as individuals age, and this might contribute to age-related declines in reproductive success (i.e., reproductive senescence; Torres and Velando 2007; Monaghan et al. 2009). Age-related declines in male mating and fertilizing success have been documented in a number of taxa (Radwan et al. 2005; Pizzari et al. 2008a; Møller et al. 2009; Dean et al. 2010; Carazo et al. 2011), and some studies have indicated a role for oxidative stress in the senescence of certain reproductive traits (Sikka 2001; Torres and Velando 2007; Pizzari et al. 2008a; Dowling and Simmons 2009; Velando et al. 2011).

Determining the extent to which oxidative stress contributes to the senescence of sexually selected traits is key to better understand the evolution of male life-history strategies and the fitness consequences of mate preferences (e.g., preference for old partners, e.g., Kokko and Lindström 1996). However, it is difficult to establish the role of oxidative stress without a pluralistic approach, which investigates how age affects the relationship between oxidative status (i.e., antioxidants and oxidative damage) and the multiple traits contributing to reproductive success. For example, in species with both pre- and postcopulatory sexual selection, males may adopt different mating strategies as they age, modifying their relative antioxidant investment to different components of reproductive effort, including sexual ornaments versus sperm quality (Preston et al. 2011). Previous studies showed that oxidative stress may negatively affect pre- and postcopulatory sexually selected traits (Metcalfe and Alonso-Alvarez 2010 and reference therein) as well as the age-related decline in such traits (Pizzari et al. 2008a; Møller et al. 2009; Velando et al. 2011; Dean et al. 2010). However, the way in which age-related changes in male oxidative status influence strategies in antioxidant allocation among different components of reproductive success remains to be elucidated. As a consequence, the contribution of such age-related patterns of antioxidant allocation to male reproductive senescence remains unresolved.

**Figure 1:**
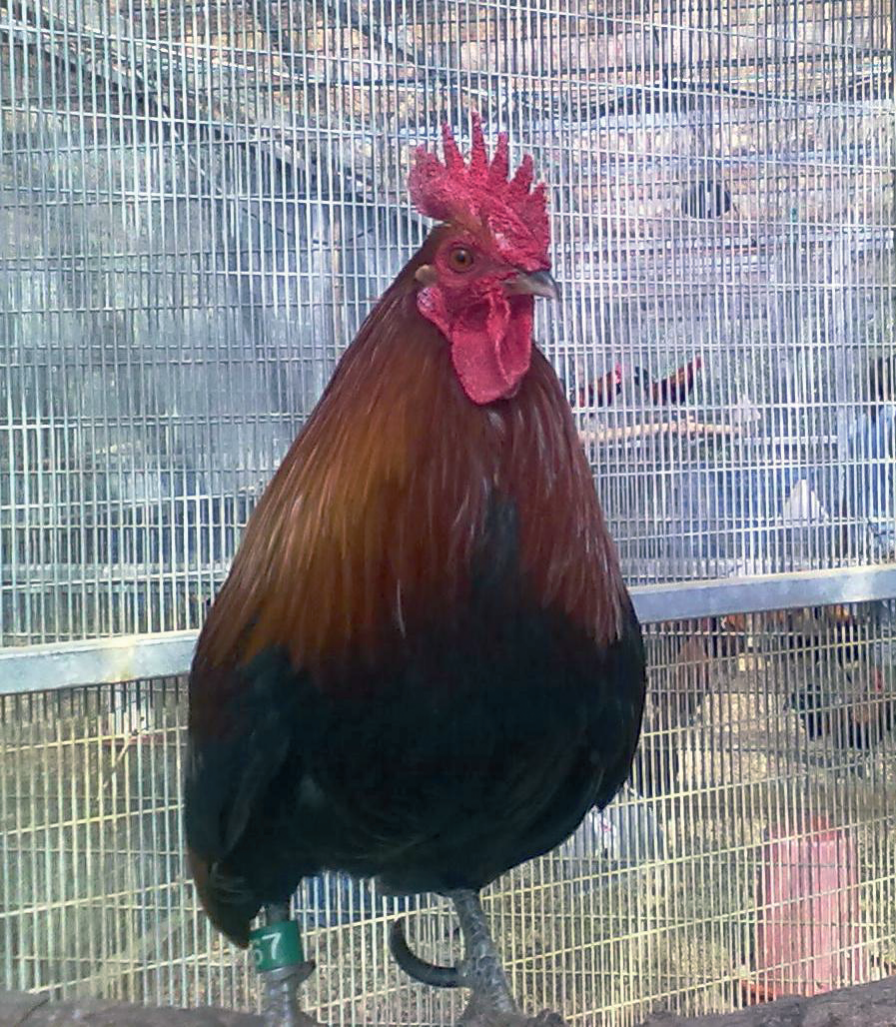
Adult red junglefowl male *Gallus gallus*. During breeding season, males show large combs, a trait important in both mate choice and male competition. Photo credit: Jose C. Noguera.

Here, we examine the relationships between oxidative status and the expression of multiple pre- and postmating reproductive traits in two-age classes of male red junglefowl, *Gallus gallu*s (Fig. 1). The red junglefowl is an appropriate system to explore age-specific changes in antioxidants allocation strategies. First, natural populations are structured in social groups (Sullivan 1991) in which multiple males compete for access to females, and male mating success is strongly influenced by male social status (Lill 1966; Johnsen et al. 2001; see also Pizzari et al. 2002). Male social status is also sexually selected after copulation, as females preferentially retain semen of dominant males (Pizzari and Birkhead 2000; Dean et al. 2011). Second, the male comb is a fleshy sexual ornament whose size is consistently associated with female preference in mate choice experiments (Zuk et al. 1990; Parker and Ligon 2003). Comb size is strongly condition-dependent in this species (Zuk et al. 1995) and has been suggested to reflect a male's oxidative stress (von Schantz et al. 1999). Finally, despite a skew in male mating success, females often mate with multiple males within a breeding season (Ligon and Zwartjes 1995), and sperm remain viable within the female sperm storage tubules for approximately 2 weeks (Etches 1996), creating a risk of sperm competition. Experiments in the domestic fowl, *G. g. domesticus*, indicate that sperm competition favors large inseminations (Martin et al. 1974), and particularly ejaculates with prolonged sperm motility and viability (Pizzari et al. 2008b). A significant proportion of inter- and intramale variation in sperm motility and viability in this species appears to be determined by seminal fluid, the nonsperm physiological component of an ejaculate (Pizzari et al. 2007; Cornwallis and O'Connor 2009). Importantly, male social status, comb size, and sperm motility tend to decline with male age (Dean et al. 2010).

The aim of this study was to determine whether these patterns of senescence are associated with changes in oxidative status. Specifically, we addressed the following questions: (i) Is social status associated with plasma oxidative status in young and old males? (ii) Does comb size covary with plasma oxidative status in young and old males? (iii) Does the oxidative status of seminal fluid change with male age? (iv) Does sperm motility covary with seminal oxidative status? (v) Does sperm DNA damage covary with seminal oxidative status in young and old males?

If male traits sexually selected before and after copulation are influenced by male oxidative status in this species, one would expect that; (1) dominant males have better oxidative status than subordinates, (2) males with better plasma oxidative status have larger combs, and (3) that males with better seminal oxidative status also have higher sperm motility and reduced levels of sperm oxidative damage. Moreover, if the availability of antioxidant resources to sexually selected traits becomes limited as a male ages, three alternative scenarios can be predicted depending on the antioxidant allocation strategy adopted by old males. First, old males could experience a decline in both comb size and sperm quality. Second, old males could allocate proportionally more antioxidant resources to either comb size or sperm quality, in which case we would expect a strong decline in only one trait (sperm quality or comb size), but less so in the other trait.

## Methods

### Study population

The study was carried out in a population of red junglefowl housed at the John Krebs Field Station of the University of Oxford, during breeding (May–July 2010). We selected sexually mature males of two-age classes: six “young” (1-year-old), and 15 “old” (4-year-old). In this species, males live at least up to 5.5 years under seminatural conditions (Collias and Collias 1996), and recent data on feral populations of domestic fowl, *G. g. domesticus*, suggest that the onset of male reproductive senescence (i.e., strong decline in the number of copulations or sperm motility) occurs on average at the age of four (Dean et al. 2010). Body mass (±1 g) and tarsus length (±0.01 mm) were measured for each male. To prevent any age-related effect of copulation rate on seminal and somatic oxidative status, males did not have access to females until the end of the study.

A subset of males (*n* = 14) were kept in groups of three for at least a week prior to sampling to allow stable social hierarchies to form. Social hierarchy was determined through behavioral observations of pairwise interactions. Males that ranked top in the trio were classified as dominant and males that were either second or third were classified as subordinate.

Semen samples were collected through abdominal massage for each male in between 11:00–16:00 h, and spermatozoa and seminal fluid were separated within 15 min by centrifugation (1 min × 14,000*g* at 4°C). To standardize sperm age across all males (Reinhardt 2007; Pizzari et al. 2008b), 48 h before sampling, males were depleted of sperm reserves through abdominal massage (Burrows and Quinn 1937). A blood sample (∼250 μL) was also obtained from the brachial vein through heparinized capillary tubes. Plasma was separated from blood cells within 15 min after collection by centrifugation (10 min × 6500*g* at 4°C). All samples (seminal fluid, spermatozoa, blood plasma, and blood cells) were immediately transferred to cryovials after centrifugation and stored in liquid nitrogen during transfer back to the laboratory for storage at –80°C until their analysis (within a month). To estimate the effect of sperm oxidative status on sperm motility in old males, we collected a semen sample in an additional group of 11 old males (4-year-old). Sperm motility was measured as sperm average path velocity (sperm swimming speed: μms-1) using computer assisted sperm analysis (Sperm Class Analyzer: SCA v. 3.0.3). One microliter of semen was diluted in 50 μL of Dolbecco's modified Eagle's medium, and 5μL of this was placed on a mounted microscope slide on a heated microscope stage at 41°C and video recorded at 200× magnification. Median sperm average path velocity was calculated per ejaculate.

Comb size was measured using digital photographs of the right- and left-side of the bird's head under standardized lighting conditions and against a standard white background together with a milimetric scale (Cornwallis and Birkhead 2007). The comb area (mm^2^) in the right- and left-side photographs of each male was measured by the same person (J. C. N.) using image analysis software (analySIS FIVE) blindly with respect to male age and status, and the mean of the left- and right-side was used all further analyses.

### Measures of antioxidant capacity and oxidative damage

#### Analysis of antioxidant capacity

The antioxidant capacity of plasma (hereafter, “plasma antioxidants”) and seminal fluid (hereafter, “seminal antioxidants”) was measured using the method described by Erel (2004). Main antioxidants contributing to the assay are hydrophilic and hydrophobic antioxidants, such as –SH group of proteins, uric acid, vitamin-C, and vitamin-E. Briefly, the method consists of mixing plasma or seminal fluid (5 μL) with acetate solution and 2,2′-azinobis-(3-ethylbenzothiazoline-6-sulfonate) (ABTS), which is decolorized by antioxidant compounds according to their concentration and antioxidant capacity. The change in color was measured as the change in absorbance at 660 nm (Synergy™ 2 Multi-Mode Microplate Reader, Bio-Tek Instruments, Inc., Winooski, VT). Samples were assayed in duplicate and showed high level of repeatability (blood plasma: *r* = 0.97, F_17,18_ = 66.377, *P* < 0.001, CV = 0.06; seminal fluid: *r* = 0.84, F_29,30_ = 11.379, *P* < 0.001, CV = 0.07; as described by Lessells and Boag 1987). Levels of plasma and seminal antioxidants were expressed as millimoles of Trolox equivalent per liter.

#### Analysis of lipid peroxidation

Lipid peroxidation (level of oxidative damage to lipids) in plasma and seminal fluid (20 μL) was assessed by quantifying malondialdehydes (MDA), using high-performance liquid chromatography, according to Karatas et al. (2002), but modifying the volume of sample as described in Noguera et al. (2011). The absorbance of the eluent was monitored at 254 nm and 1,1,3,3-tetraethoxypropane (Sigma-Aldrich, St. Louis, MO) was used as external standard (calibration curves, *R*^2^ = 0.999). Plasma and seminal fluid samples were assayed in triplicate and duplicated, respectively, and showed high level of repeatability (blood plasma: *r* = 0.99, F_17,36_ = 3.694, *P* < 0.001; seminal fluid: *r* = 0.99, F_9,10_ = 21.142, *P* < 0.001). Lipid peroxidation was expressed as μg of MDA per milliliter.

#### Analysis of oxidative DNA damage

The analysis of oxidative DNA damage present in sperm samples (spermatozoa) were assessed as described in Velando et al. (2011). Basically, sperm DNA was extracted by a Chaotropic NaI-based method, as recommended by European Standars Committee on Oxidative DNA Damage (ESCODD) to avoid artifactual oxidation. The amount of isolated DNA was determined using high sensitivity fluorescent assay (Quant-iT™ High-Sensitivity DNA Assay Kit, Invitrogen, USA), and protein contamination checked. DNA damage was estimated as apurinic–apyrimidinic (AP) sites using a biotin-labeled reagent specific for the aldehyde group in the ring-open form of AP site, designated as the aldehyde reactive probe (ARP), and according to manufacturer's instructions (ARP; Oxidative DNA Damage Quantitation kit-AP sites; Cell Biolabs, San Diego, California). ARP specifically binds to AP sites in isolated genomic DNA, and the biotin molecular in ARP can then be detected spectrophotometrically at 450 nm (Synergy™ 2 Multi-Mode Microplate Reader, Bio-Tek Instruments, Inc., Winooski). The quantities of AP sites in sperm DNA samples were assayed in duplicate in a single bout (repeatability: *r* = 0.97, F_17,18_ = 73.046, *P* < 0.001) and expressed as the number of AP sites per 100,000 base pair.

### Statistical analysis

#### Age and plasma oxidative status

We studied age-related differences on oxidative status of males by fitting two separate general linear models (GLMs) with plasma antioxidants and lipid peroxidation level as response variables. In these models, age (two-level factor), male body condition (covariate), and their interaction were fitted as fixed effects. Male body condition was calculated as residuals of linear regression of tarsus length on body mass (Schulte-Hostedde et al. 2005).

#### Comb size and plasma oxidative status

To investigate whether comb size covaries with oxidative status (“antioxidant trade-off hypothesis”), and whether age reduces antioxidant availability to comb, we fitted a GLM with comb size as the response variable, and age (two-level fixed factor) and plasma antioxidants (covariate), lipid peroxidation (covariate), and their interaction with male age as covariates. To control for any possible effect of male condition on comb size, we also included male body condition and its interaction with age as covariates in the model.

#### Seminal oxidative status and male age

To further test the antioxidant trade-off hypothesis we fitted the same model as described for the above analysis (Comb size and plasma oxidative status), but this time with seminal antioxidants as the response variable.

#### Seminal oxidative status and sperm motility

We also explored whether sperm oxidative status influences sperm motility of old males by fitting a model (GLM), which included sperm motility as dependent variable, and seminal antioxidants and lipid peroxidation levels as response variables. In this case, lipid peroxidation was previously log-transformed to fit normal distribution.

#### Seminal oxidative status and sperm DNA damage

Finally, we investigated the effect of age and seminal antioxidants on sperm DNA damage by fitting a model (GLM) with age as a fixed factor, and male body condition, seminal antioxidants, and their interactions with age as covariates. Sperm DNA damage (response variable) was log-transformed to meet model requirements. We also ran the same models including male social status (two-level factor) and its interaction with male age for the subset of males (*n* = 14) in which social status was recorded.

Multicollinearity diagnostics were examined in all models by calculating the collinearity statistic tolerance and the corresponding variance inflation factor (Quinn and Keough 2002). Tolerance values ranged from 0.69 to 0.96 indicating that the degrees of multicollinearity among the independent variables were acceptable. Following Whittingham et al. (2006), full models were also reported including nonsignificant fixed effects terms, but excluding nonsignificant interactions. Models limited to significant effects (minimal adequate models) provided similar results. All models were simplified by removing nonsignificant terms (in a backward deletion procedure), starting from two-way interactions. In four cases, digital photographs of combs were not available. Discrepancies in sample sizes between some analyses reflect missing values due to insufficient volume of blood or ejaculates samples to perform the biochemical assays (i.e., seminal lipid peroxidation or DNA damage). All statistical analyses were performed using SPSS (SPSS v.18), and the significance level was set at 0.05.

## Results

### Social status and plasma oxidative status

**Figure 2:**
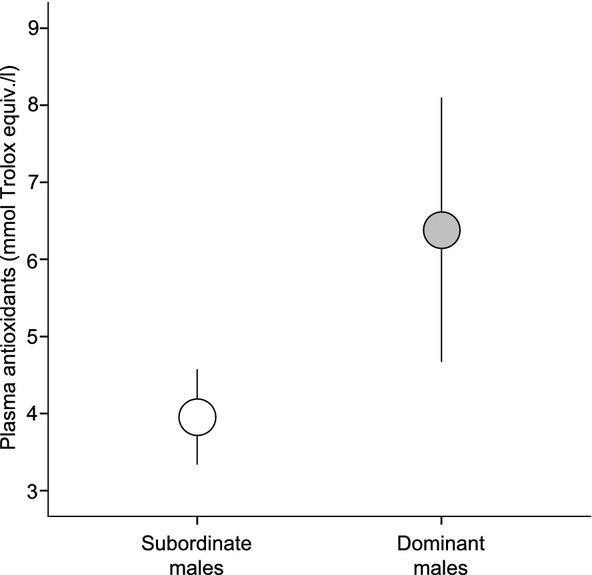
Level of plasma antioxidants of red junglefowl males, measured as mmol Trolox equivalents per liter (mean ± SE), in relation to male social status.

Oxidative status in plasma did not differ between male age classes. Old males had similar levels of plasma antioxidants and lipid peroxidation to young males (Table 1a). Similarly, plasma antioxidants and lipid peroxidation levels were not affected by male body condition or its interaction with age (Table 1a). When the model included social status, there was a significant effect of social status; dominant males had on average 33% higher levels of plasma antioxidants than subordinates (F_1,12_ = 7.172, *P* = 0.02; Fig. 2), whereas the levels of lipid peroxidation did not differ between dominant and subordinate males (estimate = –0.048, F_1,11_ = 1.952, *P* = 0.19). The interaction between age and social status did not explain variation in plasma antioxidants (estimate = 0.782, F_1,9_ = 1.653, *P* = 0.23) or lipid peroxidation levels (estimate = 0.018, F_1,9_ = 0.070, *P* = 0.79).

**Table 1:** Summary of general lineal models (GLMs) showing the influence of (a) male age on plasma oxidative status, (b) plasma oxidative status on comb size, (c) age on seminal oxidative status, (d) seminal oxidative status on sperm swimming velocity, and (e) seminal antioxidant status on oxidative DNA damage in red junglefowl (*Gallus gallus*). Full models and the models that retained only variables that caused a significant increase in deviance (minimal adequate models) are shown. Significant values in full and minimal adequate model are shown in bold

		Full model						Variables		Minimal model			
				
Dependent variable	Variables	Parameter estimate	F	df	*P*	Observed power	Effect size	Retained term	Removed term	Parameter estimate	F	df	*P*
(a) Plasma antioxidants	Intercept	2.606								2.289			
	Age	–0.123	0.067	1, 15	0.800	0.057	0.004		Age	–0.123	0.067	1, 15	0.800
	Body condition	0.001	0.405		0.534	0.092	0.026		Body condition	0.001	0.411	1, 16	0.530
									Age × Body condition	0.003	2.616	1, 14	0.128
Plasma lipid peroxidation	Intercept	0.211								0.242			
	Age	0.039	0.808	1, 15	0.383	0.134	0.051		Age	0.039	0.808	1, 15	0.383
	Body condition	–8.660e^−5^	0.882		0.363	0.142	0.056		Body condition	–3.141e^−5^	0.211	1, 16	0.652
									Age × Body condition	0.000	0.585	1, 14	0.457
(b) Comb size	Intercept	1048.984								1053.348			
	Plasma antioxidants	113.139	4.265	1, 10	0.066	0.463	0.299	Plasma antioxidants		112.732	5.695	1, 12	**0.034**
	Plasma lipid peroxidation	–2119.030	12.036		**0.006**	0.877	0.546	Plasma lipid peroxidation		–2097.537	18.132	1, 12	**0.001**
	Age	14.334	0.021		0.889	0.052	0.002		Age	12.034	0.040	1, 11	0.845
	Body condition	–0.006	0.001		0.977	0.050	<0.001		Body condition	–0.006	0.001	1, 10	0.997
									Age × Plasma lipid peroxidation	2282.606	1.528	1, 9	0.248
									Age × Plasma antioxidants	–224.511	1.749	1, 8	0.223
									Age × Body condition	0.435	0.214	1, 7	0.657
(c) Seminal antioxidants	Intercept	–3.351								–2.762			
	Age	6.686	18.144	1, 11	**0.001**	0.972	0.623	Age		6.573	12.283	1, 13	**0.004**
	Plasma antioxidants	3.373	11.069		**0.007**	0.422	0.253	Plasma antioxidants		3.512	20.522	1, 13	**0.001**
	Age × Plasma antioxidants	–4.227	43.693		**<0.001**	1.000	0.799	Age × Plasma antioxidants		–3.731	26.337	1, 13	**<0.001**
	Body condition	0.003	6.641		**0.026**	0.651	0.376		Body condition	0.003	4.409	1, 12	0.058
	Plasma lipid peroxidation	7.002	2.786		0.123	0.332	0.202		Plasma lipid peroxidation	7.002	2.786	1, 11	0.123
									Age × Body condition	–0.003	1.497	1, 10	0.249
									Age × Plasma lipid peroxidation	–32.233	0.417	1, 9	0.535
(d) Sperm motility	Intercept	57.759								64.401			
	Seminal lipid peroxidation	–24.156	3.916	1,7	0.088	0.401	0.359		Seminal lipid peroxidation	–23.955	4.671	1, 8	0.063
	Seminal antioxidants	–0.089	0.005		0.948	0.050	0.001		Seminal antioxidants	–0.089	0.005	1, 7	0.948
(e) Sperm DNA damage	Intercept	1.116								1.041			
	Age	0.115	6.273	1,14	**0.025**	0.645	0.309	Age		0.085	5.982	1, 16	**0.026**
	Body condition	–9.488e^−5^	0.832		0.377	0.136	0.056		Body condition	0.000	1.034	1, 15	0.325
	Seminal antioxidants	0.004	0.172		0.684	0.067	0.012		Seminal antioxidants	0.004	0.172	1, 14	0.684
									Age × Body condition	0.000	0.799	1, 13	0.388
									Age × Seminal antioxidants	–0.003	0.027	1, 12	0.871

### Comb size and plasma oxidative status

**Figure 3:**
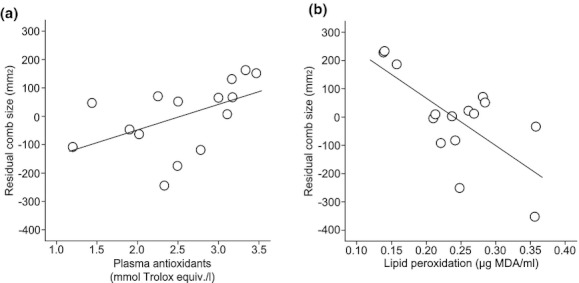
Comb size of red junglefowl males (residuals from final model after correcting by lipid peroxidation or plasma antioxidants, respectively) in relation to (a) plasma antioxidant and (b) plasma lipid peroxidation level. Lines show an adjusted linear regression (plasma antioxidants: y = 88.55x – 225.32, *r* = 0.50; lipid peroxidation: y = –1647.62x + 397.17, *r* = 0.68).

Male comb size was significantly correlated with the levels of plasma antioxidants and lipid peroxidation (Table 1b). Males with higher levels of plasma antioxidants displayed a larger comb (Fig. 3a), and this relationship was similar in both age classes (Table 1b). Moreover, the level of lipid peroxidation in plasma was negatively correlated with comb size (Table 1b; Fig. 3b). Again, this relationship was similar in both age classes (Table 1b). Male body condition, age, as well as the interaction of age with plasma antioxidants, lipid peroxidation, and body condition did not explain a significant source of variation in comb size (Table 1b). When we reran the model including social status, neither social status nor its interaction with male age explained a significant amount of variation in comb size (social status: estimate = 38.250, F_1,7_ = 0.152, *P* = 0.71; age × social status: estimate = –2970.80, F_1,3_ = 1.516, *P* = 0.31).

### Seminal oxidative status and male age

**Figure 4:**
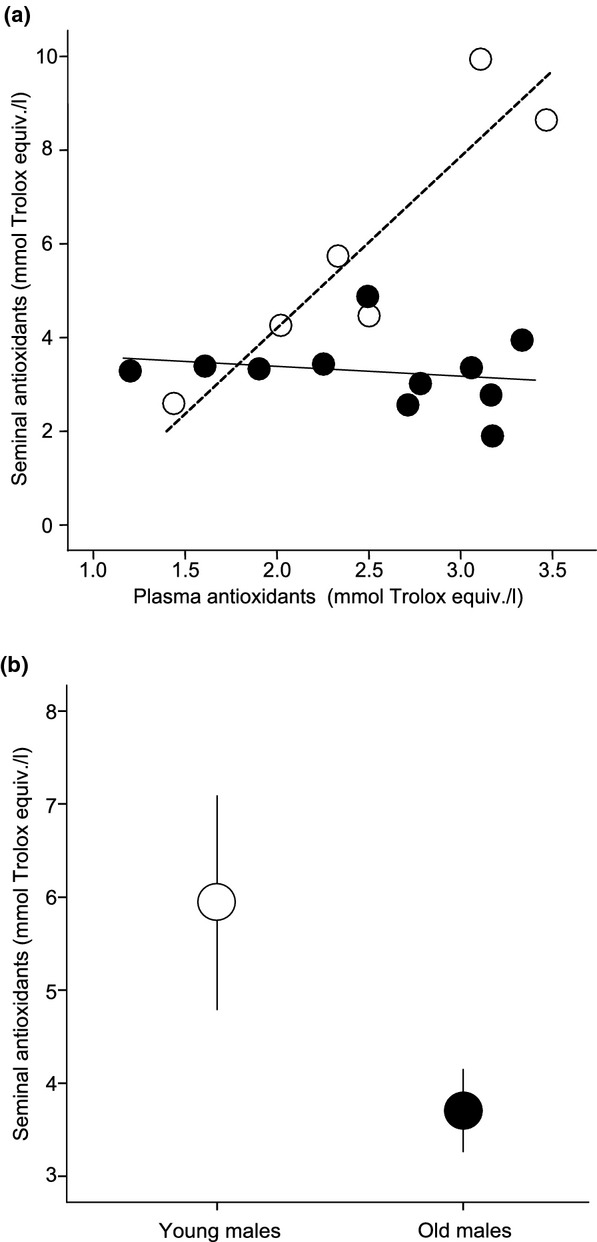
Seminal antioxidants levels in sperm of red junglefowl males. (a) seminal antioxidants in relation to plasma antioxidants (b) mean seminal antioxidants levels (±SE) in young (open circles) and old males (filled circles). Lines show an adjusted lineal regression (young males: y = 3.5x – 2.7, *r* = 0.91; old males: y = –0.2x + 3.8, *r* = 0.20).

The level of seminal antioxidants covaried positively with the levels of plasma antioxidants in young males but not in old males (Table 1c; Fig. 4a). In mean, old males had 37% less seminal antioxidants than young males (F_1,17_ = 4.976; *P* = 0.039; Table 1c; Fig. 4b). Levels of seminal antioxidants did not vary with male's body condition, lipid peroxidation in plasma, or their interactions with age (Table 1c). Social status and its interaction with age had no effect on seminal antioxidants when they were included in the model (social status: estimate = 1.373, F_1,8_ = 4.019, *P* = 0.08; age × social status: estimate = 1.871, F_1,7_ = 1.413, *P* = 0.27).

### Seminal oxidative status and sperm motility

**Figure 5:**
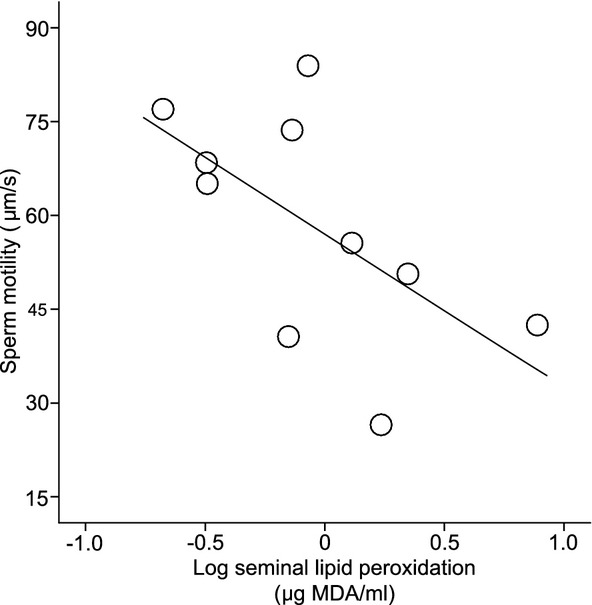
Sperm swimming velocity (average path velocity, VAP) of old junglefowls in relation to lipid peroxidation level present in the seminal fluid. Fitted line shows an adjusted linear regression (y = –23.95x + 57.3, *r* = 0.61).

Sperm motility was not correlated with seminal antioxidant levels, but there was a nonsignificant (*P* = 0.063) trend for sperm motility to decline with increasing lipid peroxidation (Table 1d; Fig. 5).

### Seminal oxidative stress and sperm DNA damage

**Figure 6:**
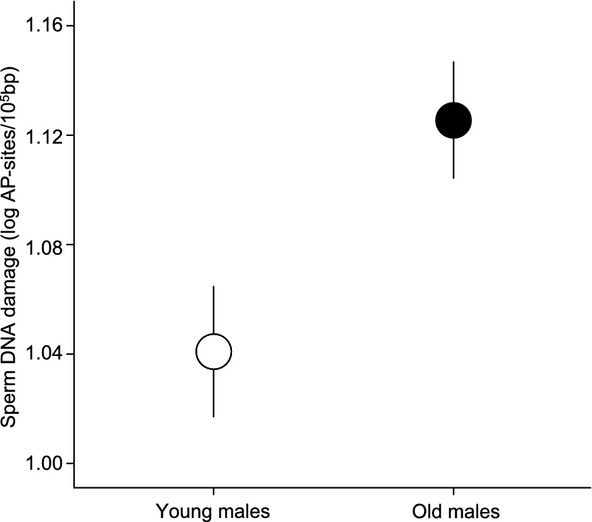
Level DNA damage in sperm of red junglefowls, measured as the number of apuric/apyrimidinic sites (mean ± SE), in relation to age classes.

Sperm DNA damage, measured as the number of AP sites, was positively correlated with male age: old males had on average 24% more AP sites in sperm DNA than young males (Table 1e; Fig. 6). Seminal antioxidants, male body condition, or their interactions with age did not explain a significant amount of variation in sperm DNA damage (Table 1e). Neither social status nor its interaction with age had a significant effect when they were included in the models (social status: estimate = –0.030, F_1,8_ = 0.782, *P* = 0.40; age × social status: estimate = 0.170, F_1,6_ = 1.939, *P* = 0.21).

## Discussion

In this study, we examined the expression of multiple pre- and postmating reproductive traits in relation to age-related changes in oxidative status. Both male social status and comb size were strongly associated with plasma oxidative status, and there was a nonsignificant tendency for sperm motility to be associated with seminal oxidative status. In addition, we found that young and old males differed in patterns of plasma and seminal antioxidant levels. Levels of seminal antioxidants increased with levels of plasma antioxidants in young males, but remained low and independent of the level of plasma antioxidants in old males, which also accumulated more oxidative damage in their sperm DNA.

Our results show that dominant males had substantially higher plasma antioxidant levels than subordinates, irrespective of age without suffering a reduction in body condition, even though dominant males spend less time feeding and resting than subordinates (Pizzari 2003; Cornwallis and Birkhead 2008). In addition, as predicted and consistent with the “antioxidant trade-off” hypothesis (von Schantz et al. 1999), we found that males with higher level of circulating antioxidants and lower lipid peroxidation had larger combs. Comb size depends on the integrity of hyaluronic acid molecules, the main component responsible for comb viscousity (Laurent and Fraser 1992; von Schantz et al. 1999). Hyaluronic acid is depolymerized by free radicals, losing viscosity, and water content (Hawkins and Davies 1996). Thus, our results suggest that males with better oxidative status are able to invest more antioxidants into sexual signaling (comb size). In addition, both male social status and comb size are strongly testosterone dependent in the fowl (Ligon et al. 1990; Johnsen and Zuk 1995; Zuk et al. 1995; Parker et al. 2002), indicating that oxidative status might be related to testosterone levels. If testosterone has pro-oxidant effects (Isaksson et al. 2011 and references therein), the present results might suggest that some males are able to sustain higher levels of oxidative stress and achieve high status and/or develop large combs compared with other males. An alternative explanation is that testosterone might in fact facilitate the bioavailability of some kind of antioxidant compounds in this species, as has been observed in adult birds by other studies (Blas et al. 2006; but see also Noguera et al. 2011). Consistent with the latter scenario, in our study population, neither plasma antioxidants decreased nor lipid peroxidation increased in dominant males, as expected, if testosterone had increased the production of pro-oxidant molecules. Thus, the testosterone-mediated effect on bioavailability of antioxidant resources might explain why dominant and/or large-combed males, with high levels of testosterone, had elevated level of plasma antioxidants. Together, these results indicate that social dominance and large combs are likely to be reliable indicators of male condition, consistent with previous work indicating the condition dependence of both traits in this species (Zuk et al. 1990, 1995; Pizzari 2003; Cornwallis and Birkhead 2008). Therefore, our results are broadly consistent with the view that oxidative stress may be a proximal mechanism underlying the honesty of sexually selected signals (von Schantz et al. 1999; Torres and Velando 2007; Monaghan et al. 2009; Metcalfe and Alonso-Alvarez 2010).

The results of our study also suggest a marked age-specific pattern in oxidative status. Aging organisms are more vulnerable to oxidative stress probably because their antioxidant systems and repair mechanisms become less efficient with age (Sohal and Weindruch 1996; Finkel and Holbrook 2000). Thus, this physiological senescence may impose a constraint to divert antioxidant resources in other functions than somatic maintenance at old ages (Finkel and Holbrook 2000; McNamara et al. 2009). Accordingly, old males had similar somatic oxidative status (i.e., plasma antioxidants and lipid peroxidation) than young males, suggesting similar investment in somatic maintenance. In contrast, old males showed lower levels of seminal antioxidants than young males. Moreover, seminal antioxidants correlated with plasma antioxidants in young but not in old males. Importantly, comb size did not differ between young and old males, a sexually selected trait that correlated with antioxidant availability. Three alternative mechanisms can explain these patterns. First, this might reflect a phenotypically plastic response whereby aging males strategically mobilize antioxidant resources toward somatic maintenance away from seminal fluid while maintaining their investment in comb size. Interestingly, even at old ages, males can be socially dominant and therefore monopolize access to females (Dean et al. 2010). This response might represent a reproductive restraint strategy (McNamara et al. 2009), with individuals restraining their reproductive effort to slow down somatic damage accumulation as they age (McNamara et al. 2009). In this case, the reduction in seminal antioxidants in old males may not (or not wholly) be a direct effect of aging, but may instead reflect a strategy aimed at slowing down somatic damage accumulation at the time that males continue to have mating opportunities. Second, the present results might reflect inter-male variation in fixed life-history strategies. In this scenario, males with more limited antioxidant investment in ejaculates are more likely to survive to old age, and as a result, our group of “old” males might be overrepresented by such males. Finally, the differences in patterns of oxidative status detected between “young” and “old” males might reflect random variation between cohorts. Because only one cohort of birds was used in each age category, we cannot rule out this possibility. Nonetheless, this result should be taken with caution because of the small sample size in the group of “young” males. Future work should seek to distinguish between these scenarios through the longitudinal analysis of multiple cohorts.

Traditional good genes models of mate choice predict the evolution of female preferences for old males (reviewed in Brooks and Kemp 2001). Nevertheless, it has been recently shown that male senescence may act as potential source of sexual conflict (e.g., Dean et al. 2010; Carazo et al. 2011; Velando et al. 2011). For example, in feral populations of domestic fowl, potential sexual conflict arises when old males are able to achieve dominant status and therefore monopolize sexual access to females, but are unable to fertilize all their eggs (Dean et al. 2010). In this study, we showed that old males do not differ from young males in terms of comb size, but suffer from worse oxidative status of seminal fluid. Furthermore, we also found a non-significant negative relationship between seminal lipid peroxidation level and sperm motility, a trait closely related to male fertility in fowl (Froman et al. 1999; Pizzari et al. 2008b). Similar results have been recently reported in other avian models (Helfenstein et al. 2010; Losdat et al. 2011). Old males had not only lower level of seminal antioxidants but also higher sperm DNA damage than young males. Similarly, it has been recently found that sperm DNA damage increases with age in the blue-footed booby, *Sula nebouxii* (Velando et al. 2011). Seminal antioxidants prevent oxidative damage in sperm (Poiani 2006; Velando et al. 2008), thereby increasing its fertilizing efficiency (Velando et al. 2008). The accumulation of oxidative damage in the sperm of older males could contribute to the age-related decline in reproductive success observed in a range of bird and mammal species (Kidd et al. 2001; Møller et al. 2009; Dean et al. 2010). Importantly, these results challenge the traditional view that germ cells are adequately protected from DNA-damaging agents and constantly rejuvenated (Kirkwood 1977; Vijg 2007). Unexpectedly, sperm DNA damages did not correlate with seminal antioxidants, but note that measures of oxidative damage informs about past exposure to high levels of oxidative stress, whereas our antioxidant analysis provided information on standing antioxidants defenses at the moment of sampling. Consequently, females mating preferentially with old males may pay a cost in terms of reduced fertility (Carazo et al. 2011; Dean et al. 2010) or viability of their young (Pizzari et al. 2008a). Our results open up the possibility that sexual conflict arising over male reproductive senescence could be at least in part, modulated by age-specific patterns of antioxidant levels.

To summarize, we found evidence of a possible role of oxidative stress as a proximal mechanism involved in the evolution of male investment in sexually selected traits. The extent to which age-specific antioxidant allocation patterns are modulated by environmental and genetic factors underpinning male's oxidative status remains to be explored.
